# Supplementary material for: From the betweenness centrality in street networks to structural invariants in random planar graphs
Source: Nat Commun. 2018 Jun 27;9:2501. doi: 10.1038/s41467-018-04978-z (PMC6021391; doi:10.1038/s41467-018-04978-z)
Supplement: Supplementary file 1 — Supplementary Information [file 41467_2018_4978_MOESM1_ESM.pdf]

Supporting Information

From the betweenness centrality in street networks to structural invariants in random planar graphs

Alec Kirkley, Hugo Barbosa, Marc Barthelemy, Gourab Ghoshal

Supplementary Note 1: Data

For each city we extracted the geospatial vector data from the OpenStreetMaps (OSM) database [1] of the roads within a bounding box circumscribing an area of 30 km radius from the city centers. The 30km radius was chosen to encapsulate both high density urban regions and more suburban regions with fewer, longer streets. After aggregating all the raw shapefile data, we populated the Rtree data structure with the linestring (a collection of latitude/longitude coordinates approximating the contour of the street) geometry of each street.

Then, for each street, we found the other streets intersecting it using the Rtree indexing, and cut the street into separate segments at each intersection point, adding a node at each of these points. Latitude and longitude coordinates of all nodes were projected onto global distances using the Mercator projection, and then an edge was added between nodes adjacent along a given street, with a weight equal to the Euclidean distance between the nodes. After searching through all streets, and checking for connectivity, the street networks were complete. The type of each street, classified into various categories by OSM ('Motorway', 'Primary', 'Service', etc.), was then added as an attribute to each edge, and two versions of the street network were created for each city. For each city, the entire street network was created, and in addition, a "refined" street network was created to approximate the network of high congestion streets, where only edges classified as primary, secondary, tertiary, highways, or service roads were kept, and all others were pruned, then the giant component of the resulting network was kept. All analyses were performed on the entire street network, except for the randomized cities analyses (i.e. randomizing the weights, rewiring the edges, etc), which were done on the filtered street networks for computational tractability. Descriptive statistics for the entire street networks of individual cities are shown in Supplementary Table 1.

It is noteworthy that in our data, individual roads are represented as single edges, regardless of the number of traffic lanes. Roads with two or more roadways with a physical barrier separating the traffic directions (e.g., divided highways and expressways) will have one edge for each physical roadway.

Supplementary Table 1: Statistics of the street networks sorted by number of nodes N (i.e., intersection). The total length ℓ corresponds to the sum of the lengths of all streets within a convex hull of area A.

City	Nodes N	Edges e	Length ℓ (km)	Area A (km^2)	Density ρ
Tokyo	612418	976040	82586.30	6552.04	93.47
Moscow	307472	482217	61391.85	11562.73	26.59
Nagoya	300588	496495	57990.36	5891.78	51.02
Osaka	292855	469333	47827.03	5968.06	49.07
Paris	279072	425108	57285.37	8911.36	31.32
Milan	201029	299564	38929.31	8412.68	23.90
Berlin	198498	306742	49006.85	10027.52	19.80
Washington DC	183687	276391	35296.45	6464.47	28.41
São Paulo	180843	283349	32579.38	5619.28	32.18
New York City	178120	288278	43137.88	6729.33	26.47
Madrid	177403	273342	33647.67	6119.91	28.99
Houston	175524	270779	34352.18	5278.51	33.25
Delhi	174732	267204	30124.32	6127.09	28.52
Los Angeles	166993	268304	38984.48	4866.74	34.31
Alexandria	162753	244978	31964.20	6371.30	25.54
Mexico City	158528	254762	27406.55	4436.46	35.73
Chicago	157740	258044	34761.24	3992.69	39.51
Toronto	156919	248099	27457.24	4307.80	36.43
Phoenix	153846	235348	32294.83	6097.68	25.23
Hyderabad	151131	231787	19793.77	4471.25	33.80
Istanbul	149511	235069	24757.37	4127.72	36.22
Buenos Aires	138245	241717	29794.35	3063.27	45.13
Philadelphia	122916	192174	31816.90	6252.88	19.66
Khartoum	122634	200241	16405.95	2972.44	41.26
Manila	118712	178773	16436.52	3031.90	39.15
Boston	118573	177186	28054.72	6991.56	16.96
Barcelona	110982	172526	23751.22	4073.86	27.24
London	105198	139541	25931.41	9401.36	11.19
Lima	99750	160214	13356.04	1841.42	54.17
Riyadh	98569	151902	20417.77	3800.11	25.94
Atlanta	92148	131969	23421.24	5949.59	15.49

Continued on next page

City	Nodes N	Edges e	Length ℓ (km)	Area A (km^2)	Density ρ
Kuala Lumpur	89879	133348	16412.21	3777.69	23.79
Rome	86374	129451	19242.76	6093.85	14.17
San Francisco	85635	135493	18801.50	4855.68	17.64
Sydney	82870	123436	17758.48	3273.63	25.31
Rio De Janeiro	82808	129256	15711.31	2956.19	28.01
Johannesburg	78377	121053	21198.68	5231.78	14.98
Jakarta	74128	112467	12520.81	2484.23	29.84
Taipei	74105	118000	15001.50	3834.81	19.32
Monterrey	73981	117784	12812.46	3724.65	19.86
Bangalore	73759	112712	13753.09	3966.04	18.60
Bogota	73648	117684	11133.31	3927.02	18.75
Miami	72411	115085	15548.93	2186.57	33.12
Bangkok	71582	102908	16316.54	4170.52	17.16
Cairo	70777	109185	16406.14	5653.43	12.52
Guadalajara	70145	113418	12226.81	5873.67	11.94
Shenzhen	65286	101370	14927.35	3810.12	17.13
Dubai	62559	91822	12126.64	2478.23	25.24
Hong Kong	62451	96059	11831.31	2716.85	22.99
Ankara	61133	95797	13571.78	5673.95	10.77
Tehran	57177	88127	12898.16	4411.81	12.96
Cape Town	52096	78827	10794.67	2460.27	21.17
Shanghai	50049	82637	19566.98	5539.79	9.03
Chennai	49278	74444	8786.05	2181.86	22.59
Baghdad	48271	75255	10837.62	4839.89	9.97
Santiago	43001	64873	18578.85	6824.06	6.30
Yangon	40840	64890	7689.51	3418.27	11.95
Kolkata	38924	57162	7258.99	3663.49	10.62
Ho Chi Minh City	38311	58902	9277.30	3679.66	10.41
Guangzhou	35921	57460	14606.66	5447.64	6.59
Luanda	35468	57329	7934.13	2101.68	16.88
Mumbain	32720	49535	7182.38	2772.80	11.80
Singapore	29756	44640	5317.58	777.07	38.29
Lahore	28008	43723	7509.84	4821.33	5.81
Surabaya	26420	39506	5467.28	2964.76	8.91
Abidjan	24499	37922	4564.90	2713.18	9.03
Melbourne	22287	33817	5789.36	3330.35	6.69
Kinshasa	21711	35563	4108.92	1624.39	13.37
Accra	21333	32060	5346.94	2062.73	10.34
Dar es Salaam	20754	31061	3564.65	2073.43	10.01
Dongguan	19294	31452	10738.15	5769.56	3.34
Lagos	18936	28066	5084.47	2406.29	7.87
Xian	18925	30592	12378.79	7923.66	2.39
Nanjing	17500	28518	9911.60	6192.78	2.83

Continued on next page

City	Nodes N	Edges e	Length ℓ (km)	Area A (km^2)	Density ρ
Nairobi	17040	24463	5529.59	3184.32	5.35
Bandung	16755	24529	3715.87	3939.03	4.25
Wuhan	16568	26508	8629.57	6446.66	2.57
Pune	16173	23905	4800.26	3905.53	4.14
Tianjin	15461	25641	10362.45	7058.71	2.19
Hanoi	14864	22934	5505.63	4592.05	3.24
Hangzhou	14829	24512	8933.17	5644.03	2.63
Kabul	14137	21517	3931.07	2919.67	4.84
Ahmadabad	13615	21046	4465.10	3988.31	3.41
Chengdu	12521	20724	7967.42	5327.72	2.35
Suzhou	12501	21104	9317.10	4952.66	2.52
Dhaka	12209	18423	3427.93	4836.00	2.52
Medan	10424	15660	2964.14	1749.25	5.96
Xiamen	9679	15652	4598.72	3758.04	2.58
Shenyang	9624	15853	7538.69	6475.61	1.49
Chongqing	8275	13232	5133.81	4740.14	1.75
Qingdao	7095	11911	4470.93	3476.07	2.04
Fuzhou	6310	9945	4519.80	4750.16	1.33
Harbin	6074	9990	4346.88	5059.06	1.20
Dalian	5654	9122	2989.32	2521.05	2.24
Kuwait	4593	6501	1826.65	4595.19	1.00
Quangzhou	3774	6189	3559.67	3248.89	1.16
Surat	3349	5020	1793.45	2635.62	1.27

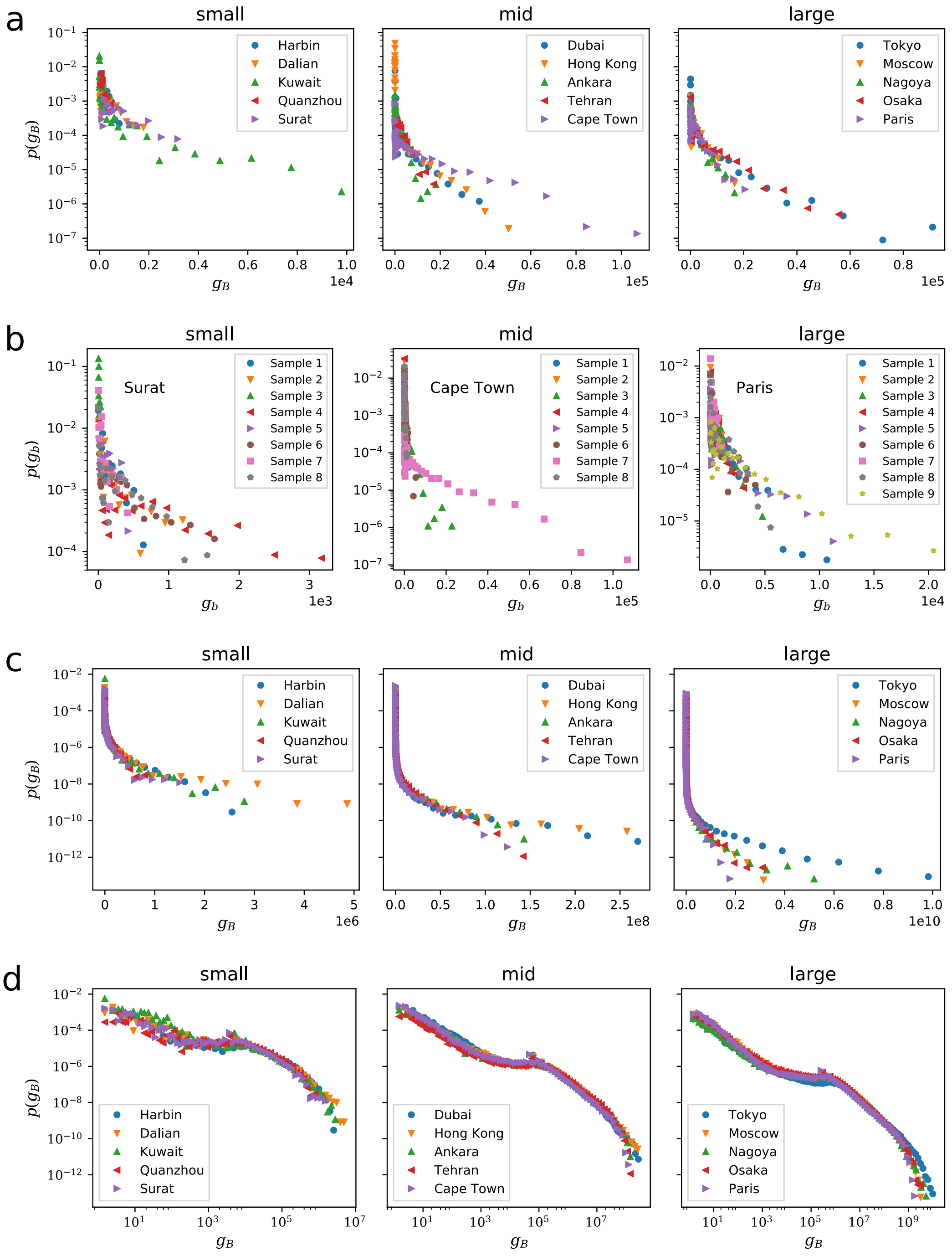

Supplementary Figure 1: Betweenness pdf's at multiple scales (a) selected one-square-mile samples from each category (log-linear). (b) Multiple one-square-mile samples within a single city picked from each category (log-linear). (c) BC of streets at full resolution $\sim$ 1000 square-miles (log-linear) and finally (d) the same in log-log scale revealing a bimodal distribution.

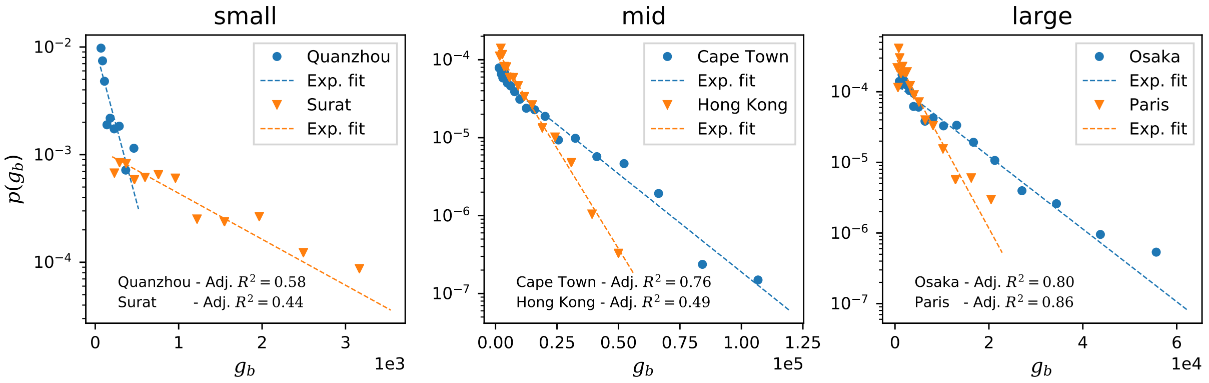

Supplementary Figure 2: Distribution of g_B for 1sq mile samples for cities of different network sizes and their corresponding exponential fits.

Supplementary Note 2: Curve fitting and verification

Curve fits were performed using the maximum likelihood procedures outlined in [2]. As we show in the main document, the tails of the BC distributions are well approximated by a truncated power-law distribution

$$p(\tilde{g}_B) \sim \tilde{g}_B^{-\alpha} e^{-\tilde{g}_B/\beta}. \tag{1}$$

Supplementary Figure 3 shows the tail of the BC distributions with their corresponding fit lines whereas Supplementary Table 2 shows the results of the curve fits for each individual city. Additionally, we fit the tails to stretched exponentials of the form

$$p(\tilde{g}_B) \sim \tilde{g}_B^{\gamma-1} e^{-\lambda(\tilde{g}_B)^{\gamma}}. \tag{2}$$

Fits for γ revealed a tightly peaked distribution near $\gamma \sim .3$ for all cities. This indicates that regardless of the exact functional form of the decay, the same power law scaling exponent of ~ -1 persists throughout all cities, with some variation that gets absorbed into the functional form of the tail decay, which is consistent with calculations for the Cayley Tree with fixed branching ratio. Therefore only the results for the truncated power law are reported in the manuscript as the exact functional form of the tails is not germane to the main discussion.

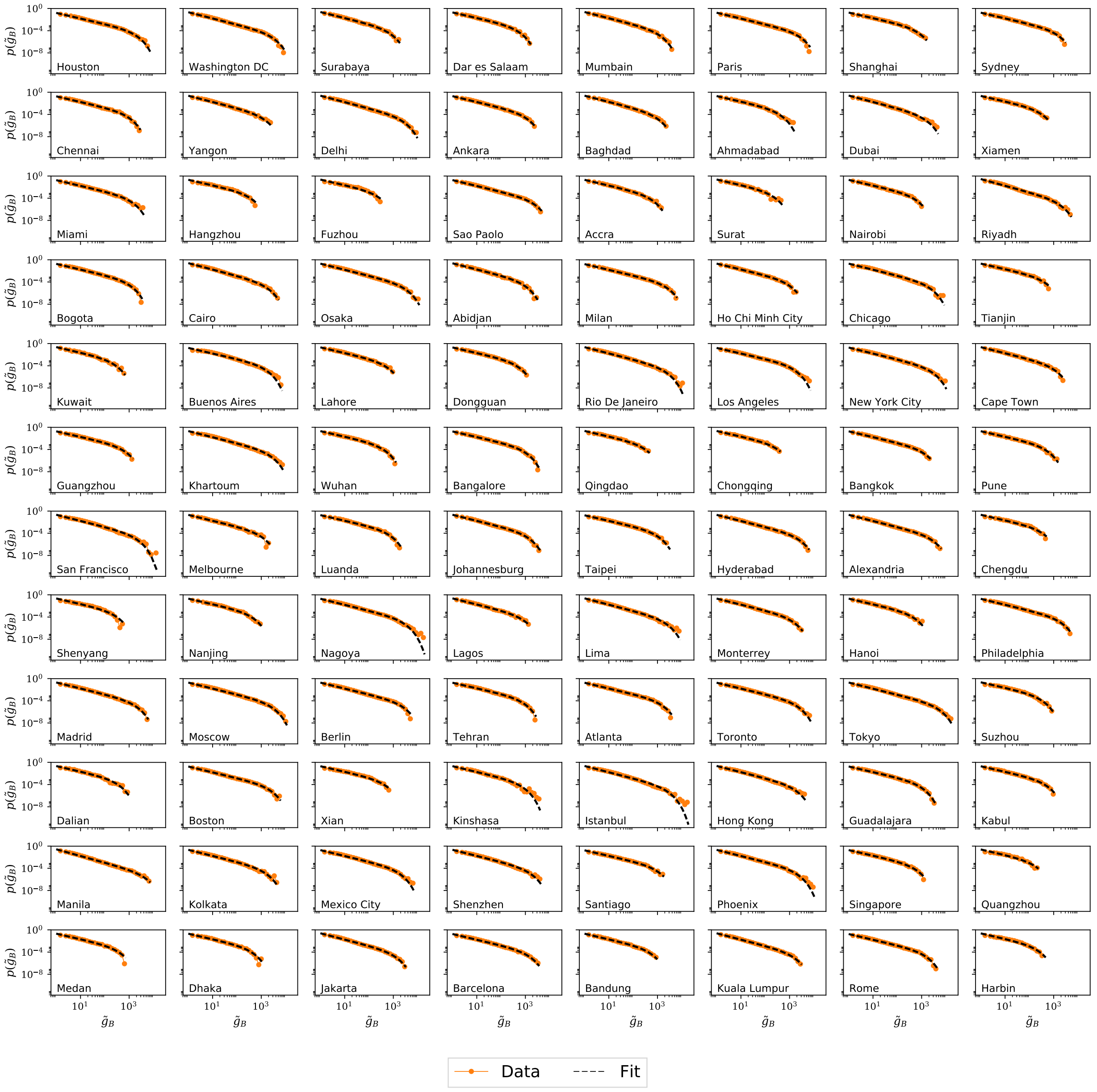

Supplementary Figure 3: Tails of the BC distributions with their corresponding truncated-power-law fits for all cities, in no particular order.

Supplementary Table 2: Parameter values for the truncated power law distributions fitted to the data.

City	α	β
Los Angeles	1.000 ± 0.002	833.56
Santiago	1.000 ± 0.003	533.34
Shanghai	1.000 ± 0.003	386.91
Tehran	1.000 ± 0.003	451.24
Taipei	1.000 ± 0.003	544.44
Guangzhou	1.000 ± 0.004	349.23
Luanda	1.000 ± 0.004	372.26
Singapore	1.000 ± 0.005	311.91
Xian	1.000 ± 0.005	246.95
Pune	1.000 ± 0.006	267.52
Wuhan	1.000 ± 0.006	222.75
Hangzhou	1.000 ± 0.006	199.18
Dongguan	1.000 ± 0.006	259.99
Tianjin	1.000 ± 0.006	228.69
Nanjing	1.000 ± 0.006	230.24
Dar es Salaam	1.000 ± 0.006	285.57
Chengdu	1.000 ± 0.007	183.08
Suzhou	1.000 ± 0.007	175.23
Kabul	1.000 ± 0.007	261.67
Dhaka	1.000 ± 0.007	228.10
Ahmadabad	1.000 ± 0.007	214.40
Medan	1.000 ± 0.008	206.62
Shenyang	1.000 ± 0.008	156.62
Xiamen	1.000 ± 0.009	166.57
Chongqing	1.000 ± 0.009	168.52
Fuzhou	1.000 ± 0.010	133.97
Qingdao	1.000 ± 0.010	159.30
Harbin	1.000 ± 0.011	121.43
Dalian	1.000 ± 0.012	181.44
Quangzhou	1.000 ± 0.014	93.07
Kuwait	1.000 ± 0.015	134.41
Surat	1.000 ± 0.016	116.08
Buenos Aires	1.001 ± 0.002	816.10
Houston	1.008 ± 0.002	1044.60
Guadalajara	1.009 ± 0.003	516.32
Bangalore	1.012 ± 0.003	534.41
Bandung	1.013 ± 0.007	268.97
Nairobi	1.015 ± 0.007	301.92
Hanoi	1.015 ± 0.007	267.13
Chennai	1.018 ± 0.003	501.82

Continued on next page

City	α	β
Bogota	1.018 ± 0.003	556.28
Lahore	1.020 ± 0.005	286.75
Sydney	1.021 ± 0.003	736.42
Accra	1.023 ± 0.006	305.32
Rome	1.024 ± 0.002	782.85
Miami	1.025 ± 0.003	679.11
Milan	1.027 ± 0.001	1289.63
Atlanta	1.029 ± 0.002	808.04
Barcelona	1.031 ± 0.002	762.74
Bangkok	1.037 ± 0.003	677.47
Kinshasa	1.044 ± 0.005	417.81
Paris	1.047 ± 0.001	1418.81
Toronto	1.047 ± 0.002	1104.54
Boston	1.049 ± 0.002	1261.84
Ankara	1.051 ± 0.003	575.70
Ho Chi Minh City	1.052 ± 0.004	502.37
Melbourne	1.056 ± 0.005	558.14
Mexico City	1.057 ± 0.002	929.95
Sao Paolo	1.059 ± 0.002	1224.01
Philadelphia	1.061 ± 0.002	1036.82
Berlin	1.068 ± 0.002	1343.56
New York City	1.073 ± 0.002	1429.50
Moscow	1.077 ± 0.001	1480.18
Madrid	1.078 ± 0.002	1030.24
Surabaya	1.082 ± 0.005	391.68
Chicago	1.083 ± 0.002	1143.91
Monterrey	1.084 ± 0.003	735.52
Nagoya	1.087 ± 0.001	1560.92
Johannesburg	1.087 ± 0.003	698.69
Lagos	1.089 ± 0.006	456.59
Kolkata	1.092 ± 0.004	823.74
Cape Town	1.092 ± 0.004	565.36
Shenzhen	1.096 ± 0.003	868.03
Osaka	1.097 ± 0.001	1700.57
Phoenix	1.097 ± 0.002	1119.66
Hyderabad	1.097 ± 0.002	1323.22
Khartoum	1.100 ± 0.002	1262.22
Tokyo	1.101 ± 0.001	2698.20
Abidjan	1.101 ± 0.005	504.54
Alexandria	1.102 ± 0.002	1438.91
San Francisco	1.107 ± 0.003	1112.27
Mumbain	1.110 ± 0.005	662.04
Hong Kong	1.111 ± 0.003	921.48

Continued on next page

City	α	β
Baghdad	1.113 ± 0.004	519.98
Washington DC	1.115 ± 0.002	1642.06
Yangon	1.117 ± 0.004	700.39
Rio De Janeiro	1.122 ± 0.003	1218.63
Istanbul	1.123 ± 0.002	1410.86
Jakarta	1.132 ± 0.003	672.21
Lima	1.139 ± 0.002	1236.50
Kuala Lumpur	1.140 ± 0.003	830.67
Delhi	1.150 ± 0.002	1393.89
Cairo	1.151 ± 0.003	957.97
Dubai	1.152 ± 0.003	783.79
Riyadh	1.205 ± 0.003	997.07
Manila	1.208 ± 0.002	2497.66

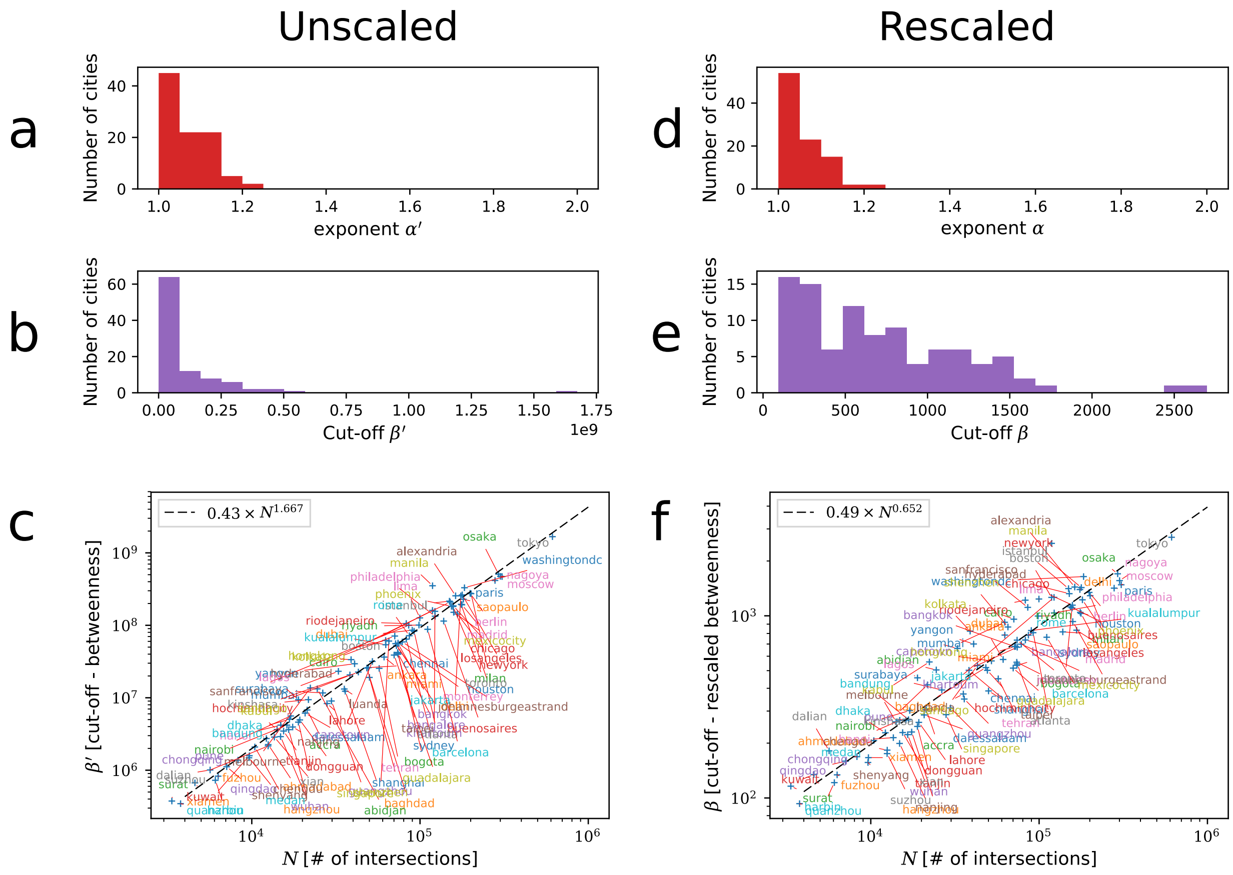

Supplementary Figure 4: Distribution of the truncated-power-law parameters for both the unscaled (a-c) and rescaled BC (d-f). (a,d) The distribution of α is almost identical in both the rescaled and unscaled BC distributions. The distribution of the exponential cut-off, β, changes dramatically from the unscaled (b) to the rescaled versions of the BC (e). These cut-offs (c,f) also show a marked dependence on system size (number of nodes)

Supplementary Figure 4 shows the distribution of power law exponents α, α' and decay exponents β, β' obtained for the tails of the betweenness distributions of all cities studied, both for the regular and rescaled betweenness. Also shown are the exponents β, β' as a function of N.

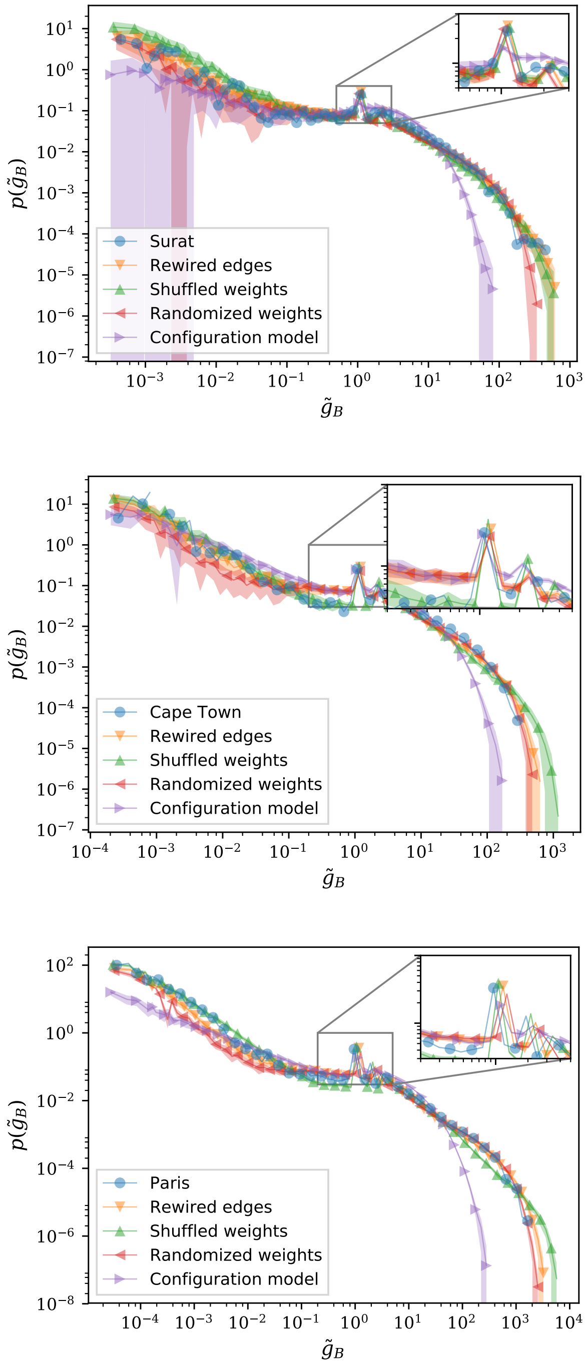

Supplementary Figure 5: The BC distribution of various random graph models described in the main text compared to the baseline distribution of representative examples of cities of different sizes. Shaded area reflects fluctuations around the average over hundred realizations of each model.

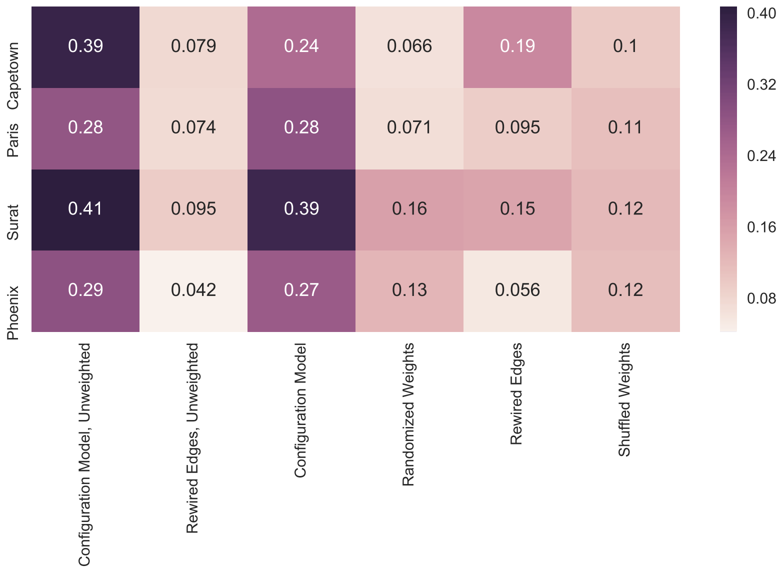

Supplementary Figure 6: Average KS statistics over all 100 realizations of each random graph model when compared to the tail of the true betweenness distribution for cities at various size scales. In all cases, the non-planar configuration random graphs exhibit the most statistical dissimilarity from the original network in the tails of their betweenness distributions.

Supplementary Figure 5 shows the betweenness distributions for the randomized versions of cities at various size scales in the same manner as what was done for Phoenix in Figure 2 in the main manuscript. The similarity in the distributions seen in that figure is replicated in these plots, and the corresponding 2-sample KS statistics for each random graph model (along with the unweighted versions of these simulations) are shown in Supplementary Figure 6 and and Supplementary Figure 7. To obtain the reported values, the KS statistics were obtained for the comparison of the actual Phoenix street network tail (nodes with betweenness above N) and the tails of each of the 100 realizations of the given random graph model, which were then averaged to get a single value. Although the comparisons are not statistically significant for random graph models of large cities, we do see statistical significance in Surat, a much smaller city of 300 nodes, as well as for random samples from larger cities of sizes up to ~ 500 nodes. The KS statistic for the comparison of the street network with its non-planar configuration model counterpart is more than double that of the next highest KS value, indicating that the constraint of planarity has a much stronger effect on the betweenness distribution than other structural perturbations.

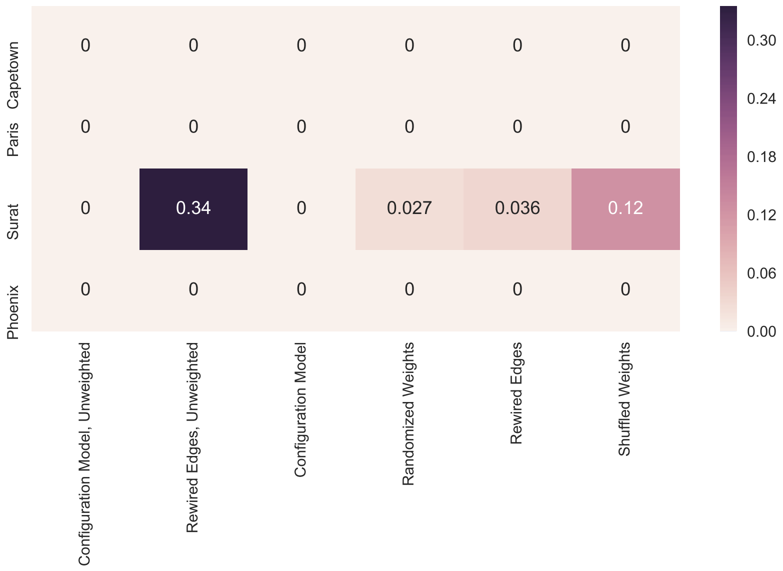

Supplementary Figure 7: Average 2-sample KS p-values over all 100 realizations of each random graph model when compared to the tail of the true betweenness distribution for cities at various size scales.

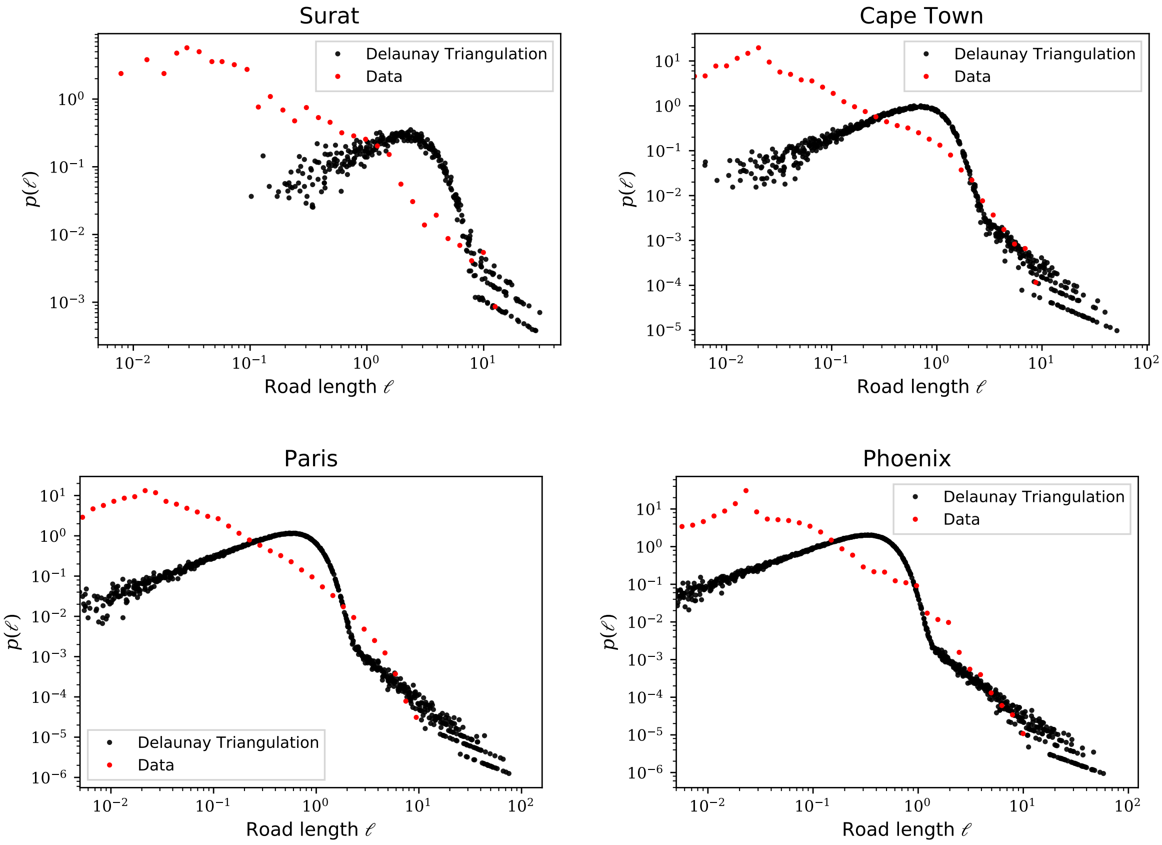

ca

Supplementary Figure 8: Distribution of road segment lengths (in km) for three selected cities of different sizes along with the length distribution of their corresponding Delaunay Triangulations.

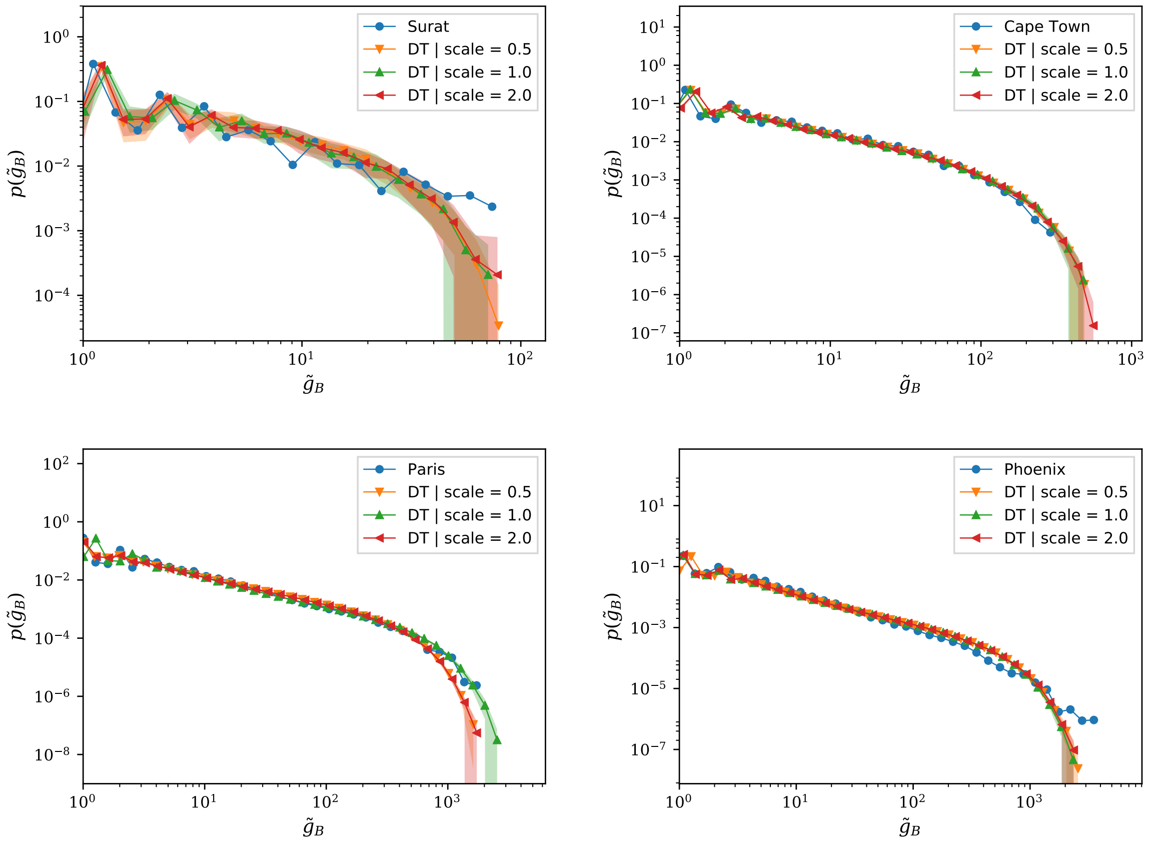

Supplementary Figure 9: Tails of the BC distributions for selected cities and their corresponding DT for different grid-sizes, having the effect of changing the area and therefore the density of nodes N/A. Shown are the results for half and twice the original areas.

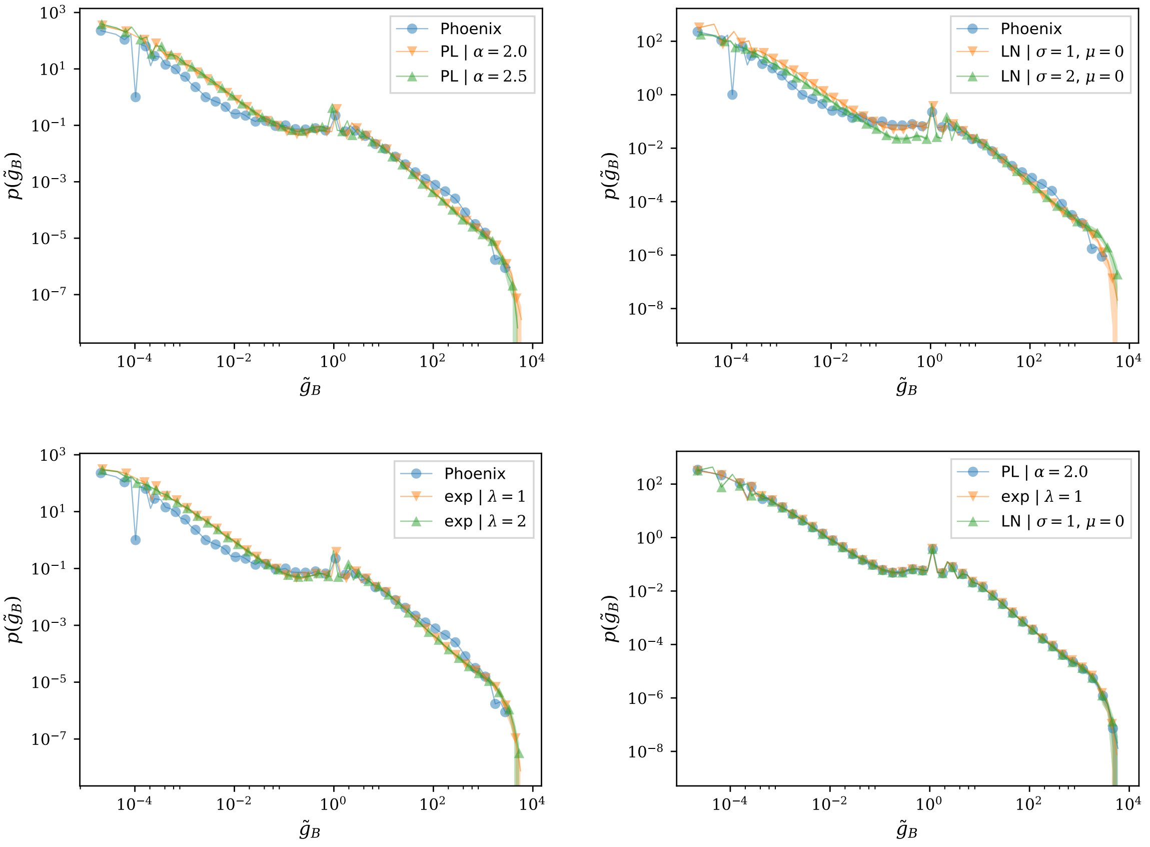

Supplementary Figure 10: Distribution of $\tilde{g}_B$ for the Phoenix street network, with edge weights generated randomly from multiple families of distributions; power law (PL), exponential (exp) and log normal (LN). The bottom right plot shows three different weight distributions leading to an identical BC profile.

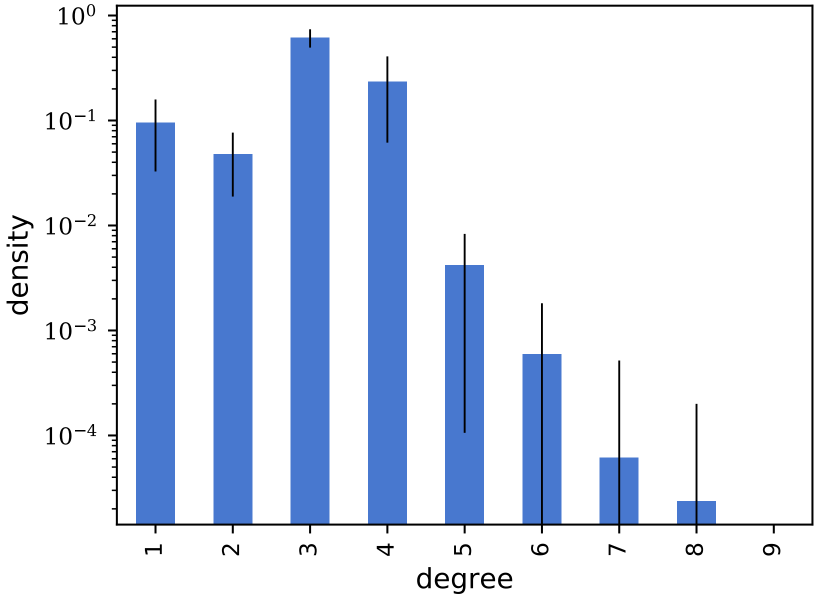

Supplementary Figure 11: Degree distribution of streets networks averaged over different cities. The length of the black line corresponds to the standard deviation across the cities.

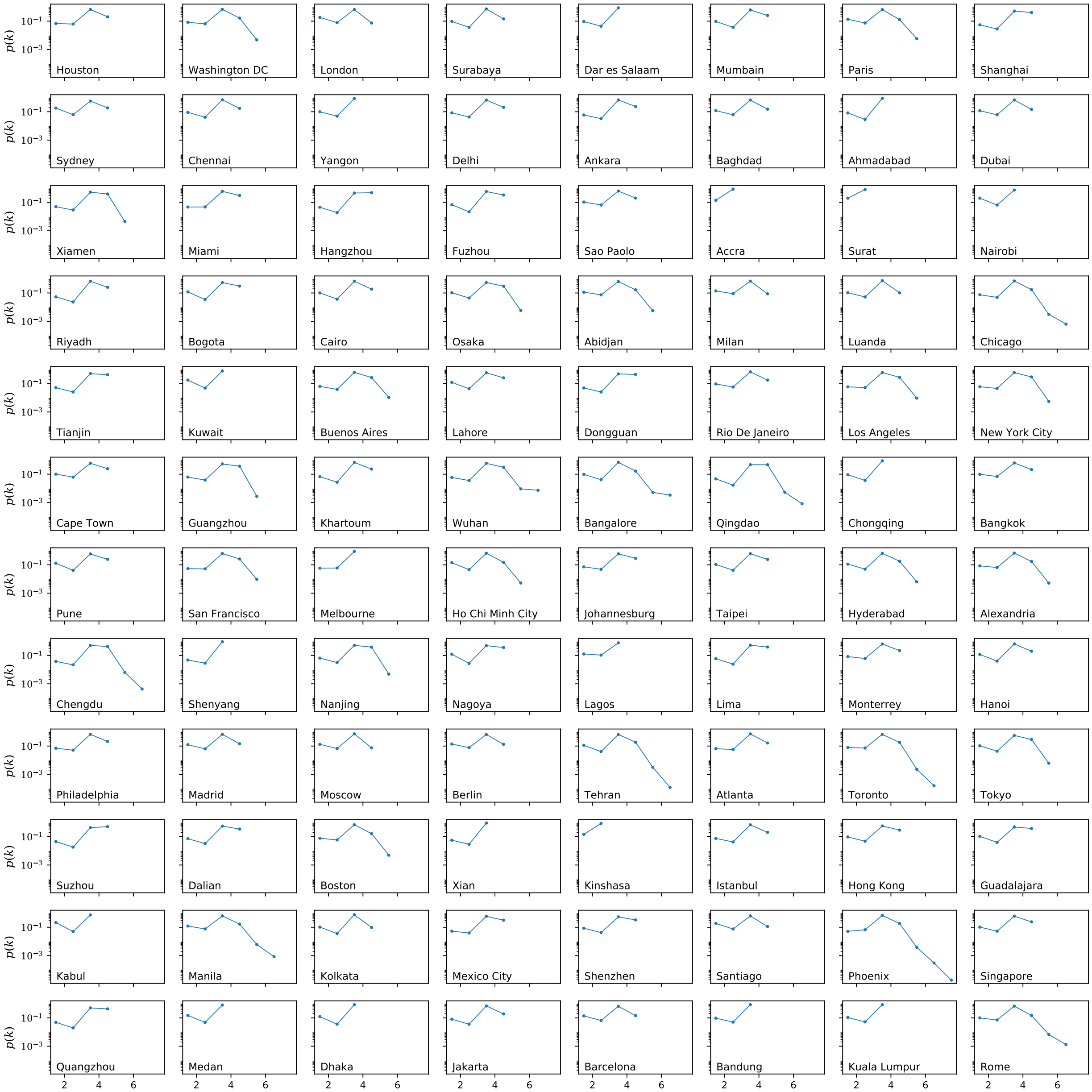

Supplementary Figure 12: Degree distribution for each individual city, in no particular order.

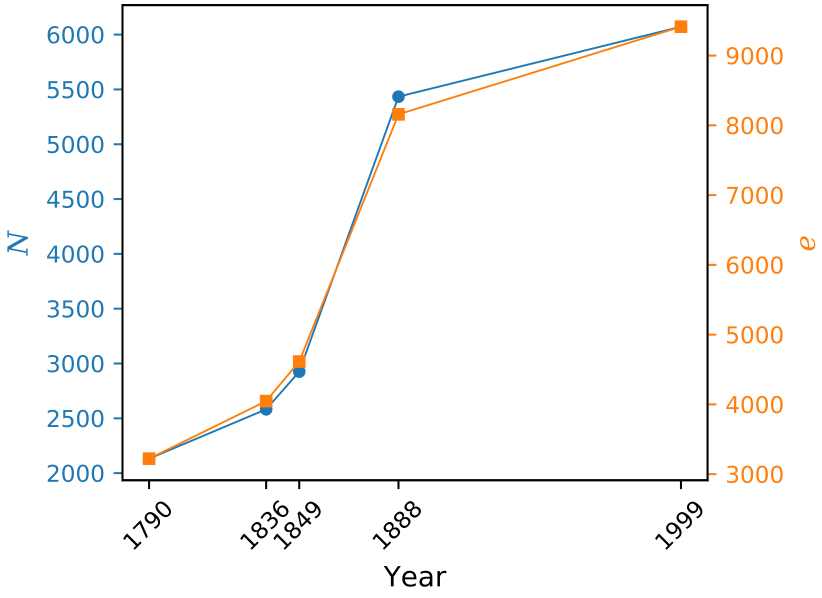

Supplementary Figure 13: Evolution of the 1789 portion of the Paris street network over a period of approximately 200 years in terms of the number of nodes N and edges e. The edge-density is roughly constant, given that nodes and edges grow at the same rate.

Supplementary Note 3: Betweenness centrality of the fastest travel routes

In addition to the full street networks, we also analyzed subsets of the networks corresponding to the fastest travel routes connecting thousands of origin and destination points in the cities. This was motivated by the fact that the fastest routes encapsulate additional dimensions of the underlying road infrastructure such as road capacities and speeds limits. The analysis was conducted on 15 cities sampled from small, mid-sized and large urban areas.

The fastest travel routes were extracted as following: for each city we generated 36 points assigned along the circumferences of circles of 2km, 5km, 10km, 15km, 20km and 30km radii from the city center and spaced at multiples of 10° and enumerated over all OD pairs by connecting the 36 points at a given radius r for a total of $6 \times \binom{36}{2} = 3780$ total OD pairs. We then queried the OpenStreetMap routing API and requested the *fastest* routes connecting each OD pair. The fastest routes are generated by the OSM routing service based on the roads' metadata such as speed limits and road types (e.g., motorway vs. residential), reflecting planning choice and route preferences.

To avoid the influence of unfeasible starting or destination points generated by our method, we discarded from our data the routes whose actual starting or destination points—i.e., the coordinates returned by the OSM API as the starting or ending points of a route—were more than 1km off from the queries point. Moreover, we also excluded those routes whose lengths are longer than $3s + 1km$, where s is the geodesic distance between the origin and destination points. This procedure is the same adopted in [3]. The roads that appeared in at least one of the remaining suggested routes were selected. The networks were then constructed in the same way as we did for the full set of streets.

Supplementary Table 3 shows the summary statistics for the fastest travel routes in comparison to the complete road data. For the largest cities, the fastest routes correspond to only a small fraction of the overall networks ($\sim 10\%$), while in small urban areas, the fastest routes encompass large fractions of the road infrastructure($\sim 40\%$). Despite this variability, the BC distributions of the fastest route networks exhibit exactly the same scaling properties seen for the full street network as seen in Supplementary Figure 14.

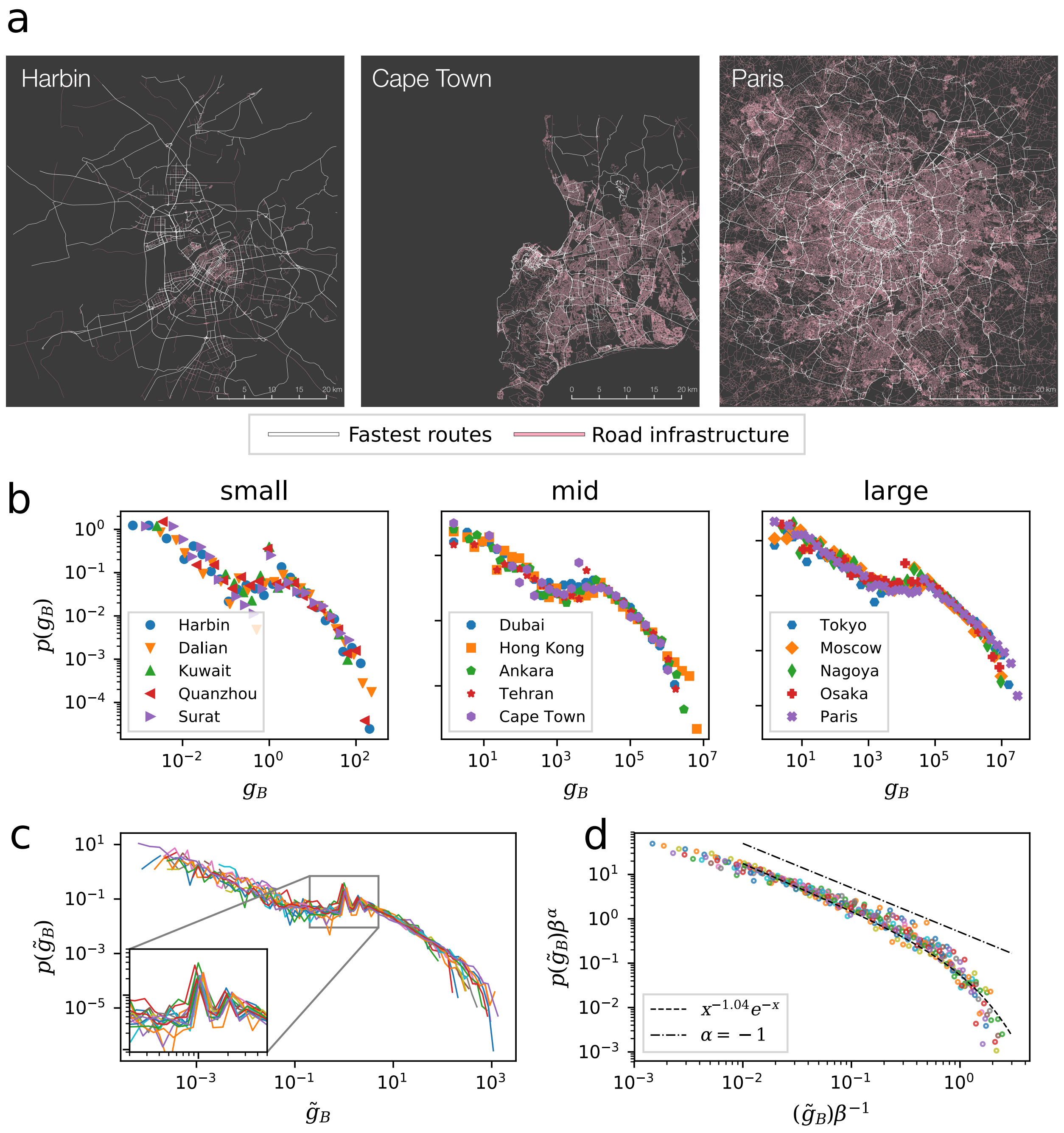

Supplementary Figure 14: Betweenness centrality of fastest travel routes. (a) The fastest route network (white) overlaid on the full street network (light red) for three different cities chosen from each class (small, mid, large) (b) The BC distribution of the fastest routes exhibit the same properties of the complete streets networks, in spite of their constituting a fraction of the original networks (Supplementary Table 3). (c) The rescaled BC distribution ($\tilde{g}_b$) collapse on to a single curve with a unique bump at $\tilde{g}_b = 1$ separating the two regimes. (d) Rescaling with respect to β results in a collapse of the tails, with an exponent $\alpha \approx 1.04$.

Supplementary Table 3: Summary statistics for streets networks lying in the fastest travel routes for 15 cities. N_{routes} corresponds to the total number of travel routes obtained for each city that lie on the fastest paths. ℓ denotes the total length of the roads systems we analyzed whereas ℓ_{routes} denotes the lengths of the roads that were part of the fastest travel routes. The ratio ℓ_{routes}/ℓ represents the fraction of the total road length corresponding to the fastest routes. V and V_{routes} represent the number of nodes in the original and in the routes networks respectively while E and E_{routes} denote the number of edges in the respective networks.

City	N_{routes}	ℓ (km)	ℓ_{routes} (km)	ℓ_{routes}/ℓ	V	E	V_{routes}	E_{routes}
Tokyo	3,540	77,087.06	5,018.89	0.07	612,418	976,040	18,991	23,930
Nagoya	3,725	52,523.96	4,618.46	0.09	300,588	496,495	11,763	15,037
Paris	3,780	46,319.14	4,551.87	0.10	279,072	425,108	22,435	27,186
Osaka	3,305	46,049.61	4,572.42	0.10	292,855	469,333	13,043	17,018
Moscow	3,710	43,845.49	3,253.94	0.07	307,472	482,217	10,587	12,314
Hong Kong	3,004	14,437.54	2,392.11	0.17	62,451	96,059	6,867	8,415
Cape Town	1,683	14,301.90	1,758.95	0.12	52,096	78,827	3,784	4,481
Dubai	2,369	14,196.70	2,285.92	0.16	62,559	91,822	4,747	6,394
Tehran	3,245	13,285.97	2,994.92	0.23	57,177	88,127	5,758	7,608
Ankara	3,326	12,632.22	2,806.72	0.22	61,133	95,797	5,805	7,650
Quanzhou	2,446	3,879.01	2,034.42	0.52	3,774	6,189	1,713	2,340
Harbin	2,582	3,806.71	1,883.63	0.49	6,074	9,990	2,106	2,840
Dalian	1,732	2,909.62	1,252.77	0.43	5,654	9,122	1,960	2,604
Surat	2,672	2,161.52	1,093.23	0.51	3,349	5,020	1,052	1,365
Kuwait	1,237	2,084.75	891.43	0.43	4,593	6,501	585	778

Supplementary References

[1] OpenStreetMap contributors. Planet dump retrieved from https://planet.osm.org . `https://www.openstreetmap.org` (2017).

[2] Clauset, A., Shalizi, C. R. & Newman, M. E. J. Power-law distributions in empirical data. *SIAM Rev.* **51**, 661–703 (2009).

[3] Lee, M., Barbosa, H., Youn, H., Holme, P. & Ghoshal, G. Morphology of travel routes and the organization of cities. *Nat. Commun.* **8**, 2229 (2017).
